# 38 Teen-Aged Life Impact Burn Recovery Evaluation: Interpersonal Interactions and Relationships Item Pool Creation Involving Teens

**DOI:** 10.1093/jbcr/iraf019.038

**Published:** 2025-04-01

**Authors:** Sophia McLaughlin, Madeleine McGwin, Khushbu Patel, Alexandra Gladstone, Yasameen Farahvash, Ludwik Branski, Tina Palmieri, Frederick Stoddard, Michael Murphy, Jeffrey Schneider, Lewis Kazis, Mary Slavin, Colleen Ryan

**Affiliations:** Shriners Children’s - Boston; Shriners Children’s - Boston; Boston Medical Center; Northeastern University; Shriners Children’s Northern California; University of Texas Medical Branch; University of California, Davis and Shriners Children’s; Harvard Medical School; Spaulding Rehabilitation Hospital, Harvard Medical School; Boston University; Spaulding Rehabilitation Hospital; Shriners Children’s - Boston and Massachusetts General Hospital; Shriners Children’s - Boston, Massachusetts General Hospital, Harvard Medical School

## Abstract

**Introduction:**

Adolescents undergo major development and social changes. However, coupling adolescence with burn injuries can complicate social interactions and relationships. The Teen-Aged Life Impact Burn Recovery Evaluation (TA-LIBRE12-19) is being developed as a burn- and age-specific outcome metric. Using the World Health Organization’s International Classification for Functioning, Disability, and Health for Children and Youth, alongside a comprehensive literature review, a conceptual framework was developed. Two overarching domains were identified: Activity and Participation. This study details the qualitative analysis of transcripts from teen burn survivors used to coproduce an item pool related to Interpersonal Interactions and Relationships, a proposed subdomain of the Participation domain.

**Methods:**

Generic and burn-specific items from legacy instruments were reviewed through binning and winnowing in previous work. Stakeholder focus groups involving teens were conducted to identify key outcomes for assessing burn recovery. Teens were asked to share their thoughts and opinions on how burns impacted their lives. Focus group transcripts were analyzed, and emergent themes were identified using NVivo software until thematic saturation was reached. Themes were assigned to existing domains or new items were created based on recurring themes raised by the teens that were not otherwise covered.

**Results:**

Previous analyses identified 1,446 items from legacy instruments possibly related to Interpersonal Interactions and Relationships. Repeated rounds of expert review resulted in 23 items. Four focus groups were conducted with eight teen burn survivors and/or their representative parents. Median age was 14.2 years (IQR 12.1-16.3), 62% of whom were female. Half of burns were flame-related, median time since injury was 6.3 years (IQR 0.6-13.7) and median burn size was 20% (IQR 0.3-6) TBSA. The focus groups resulted in 28 new items. The total Interpersonal Interactions and Relationships subdomain contains 51 items, 45.1% (n=23) from legacy instruments and 54.9% (n=28) developed from focus group input.

**Conclusions:**

Survivor input is critical in the development of the TA-LIBRE12-19 instrument. Over half of the items were newly developed from teens’ qualitative input, demonstrating the value of engaging persons with lived experience. Focus groups revealed limitations in current teen assessment tools, providing key insights for new item development.

**Applicability of Research to Practice:**

Coproduction makes TA-LIBRE12-19 a granular, precise measure of teenage burn outcomes. The next step is calibration and psychometric testing of all item pools. This will start with field-testing these items with 500 teens. Following this, a computer adaptive test will be developed.

**Funding for the Study:**

This work was funded by Foundation Funding (Grant #79136) and partial support from the National Institute on Disability, Independent Living, and Rehabilitation Research (Grant #90DPBU0008).

